# Inhibitors of the VEGF Receptor Suppress HeLa S3 Cell Proliferation via Misalignment of Chromosomes and Rotation of the Mitotic Spindle, Causing a Delay in M-Phase Progression

**DOI:** 10.3390/ijms19124014

**Published:** 2018-12-12

**Authors:** Daiki Okumura, Mari Hagino, Akane Yamagishi, Yuichiro Kaibori, Sirajam Munira, Youhei Saito, Yuji Nakayama

**Affiliations:** Department of Biochemistry & Molecular Biology, Kyoto Pharmaceutical University, Kyoto 607-8414, Japan; ky13074@poppy.kyoto-phu.ac.jp (D.O.); ky14274@poppy.kyoto-phu.ac.jp (M.H.); ky14374@poppy.kyoto-phu.ac.jp (A.Y.); kd15002@poppy.kyoto-phu.ac.jp (Y.K.); kd16015@poppy.kyoto-phu.ac.jp (S.M.); ysaito@mb.kyoto-phu.ac.jp (Y.S.)

**Keywords:** VEGFR, SU4312, Ki8751, A83-01, M phase, cell division, misalignment, spindle rotation, spindle assembly checkpoint, RO-3306

## Abstract

Cell division is the process by which replicated chromosomes are separated into two daughter cells. Although regulation of M phase has been extensively investigated, not all regulating factors have been identified. Over the course of our research, small molecules were screened to identify those that regulate M phase. In the present study, the vascular endothelial growth factor receptor (VEGFR) inhibitors A83-01, SU4312, and Ki8751 were examined to determine their effects on M phase. Treatment of HeLa S3 cells with these inhibitors suppressed cell proliferation in a concentration-dependent manner, and also suppressed Akt phosphorylation at Ser473, a marker of Akt activation. Interestingly, cleaved caspase-3 was detected in Adriamycin-treated cells but not in inhibitor-treated cells, suggesting that these inhibitors do not suppress cell proliferation by causing apoptosis. A cell cycle synchronization experiment showed that these inhibitors delayed M phase progression, whereas immunofluorescence staining and time-lapse imaging revealed that the M phase delay was accompanied by misalignment of chromosomes and rotation of the mitotic spindle. Treatment with the Mps1 inhibitor AZ3146 prevented the SU4312-induced M phase delay. In conclusion, the VEGFR inhibitors investigated here suppress cell proliferation by spindle assembly checkpoint-induced M phase delay, via misalignment of chromosomes and rotation of the mitotic spindle.

## 1. Introduction

Cell division is the process by which organelles, cytoplasm, and replicated chromosomes are separated into two daughter cells. The serine/threonine kinase Cdk1 orchestrates many proteins that ensure the high fidelity of M phase [1]. Other kinases, including Plk1 [2], Aurora kinases [3], and NEK family kinases [4], are also important M phase regulators and have been extensively investigated. However, not all regulators of M phase progression have been identified. Over the course of our research, we attempted to screen small molecules to identify molecules affecting M phase progression, and found that some kinase inhibitors targeting the vascular endothelial growth factor (VEGF) receptor (VEGFR) inhibit M phase progression.

Receptor-type tyrosine kinases (RTKs) are crucial signaling molecules in cells, and their oncogenic mutation causes malignant transformation. Indeed, increased RTK activity due to overexpression and oncogenic mutation has been demonstrated in a wide variety of cancer cells. VEGFR is mainly expressed in endothelial cells, and is one of the more extensively investigated RTKs. VEGF/VEGFR signaling is the most important regulatory factor of angiogenesis and mediates various cellular responses, including vascular permeability and cell survival, migration, and proliferation in blood vascular endothelial cells [5]. Although VEGFR-1 and VEGFR-3 are able to bind VEGF, VEGFR-2 is primarily responsible for VEGF-potentiated angiogenesis and cell proliferation [5]. When VEGF binds to VEGFR, VEGFR dimerizes and autophosphorylates its tyrosine residues. The phosphorylated tyrosine residues in the intracellular domain are then recognized by the Src homology 2 domain of the proteins, which stimulate downstream signals including the phospholipase Cγ (PLCγ)–ERK1/2 pathway, the PI3K–AKT pathway, and Src. Phosphorylated tyrosine residue of VEGFR at 1173 recruits PLCγ; PLCγ is activated and catalyzes generation of inositol 1,4,5-triphosphate (IP3) and diacylglycerol (DAG). This regulates the RAF1–MEK–ERK1/2 cascade via activation of PKCβ2. This pathway plays a central role during vascular development. PI3K–Akt pathway is crucial for cell survival. PI3K is indirectly activated downstream of VEGFR via either SRC and VE-cadherin or AXL. SRC activation depends on phosphorylation of tyrosine residue at 949 and regulates vascular permeability through phosphorylation of its substrates including cytoskeletal components [5]. In addition to endothelial cells, VEGFR is expressed in breast [6], bladder [7], colorectal [8,9], and gastric [10] cancers; it is an attractive target for cancer chemotherapy.

The selective inhibitor of VEGFR-2, SU4312, which has been designed and synthesized as a novel class of tyrosine kinase inhibitors, is highly specific against VEGFR-2 (IC_50_ of 0.8 µM) in ligand-dependent autophosphorylation assays [11]. SU4312 inhibits VEGF-dependent angiogenesis (IC_50_ of 1.8 µM) [12], and is able to reduce the proliferation of multiple myeloma and leukemic cells at 10 µM in vitro [13]. Meanwhile, Ki8751, which has been found to be a specific VEGFR-2 inhibitor through synthetic modifications of a lead compound and structure–activity relationship studies, inhibits VEGFR-2 phosphorylation in intact-cell assays (IC_50_ of 0.9 nM) and, at slightly higher concentrations, PDGFR family members [14]. It also suppresses the proliferation of human umbilical vein endothelial cells at 1 nM in vitro, and inhibits cancer cell growth in a nude mouse xenograft model [14]. The TGF-βR1 (ALK5) kinase inhibitor A83-01, which was developed as a TGFβ inhibitor, also inhibits VEGFR with a similar potency to ALK5 kinase; A83-01 at 0.1 µM inhibits VEGFR kinase activity by more than 50% [15]. In the present study, we analyzed the effects of these kinase inhibitors on M phase progression. We found that they caused a delay in M phase progression at a concentration around their IC_50_ values. A cell cycle synchronization experiment and time-lapse imaging revealed that the delay in M phase progression was caused by misalignment of chromosomes and rotation of the mitotic spindle. These results suggest possible mechanisms that may underlie the suppression of cell proliferation by these inhibitors.

## 2. Results

**Figure 1:**
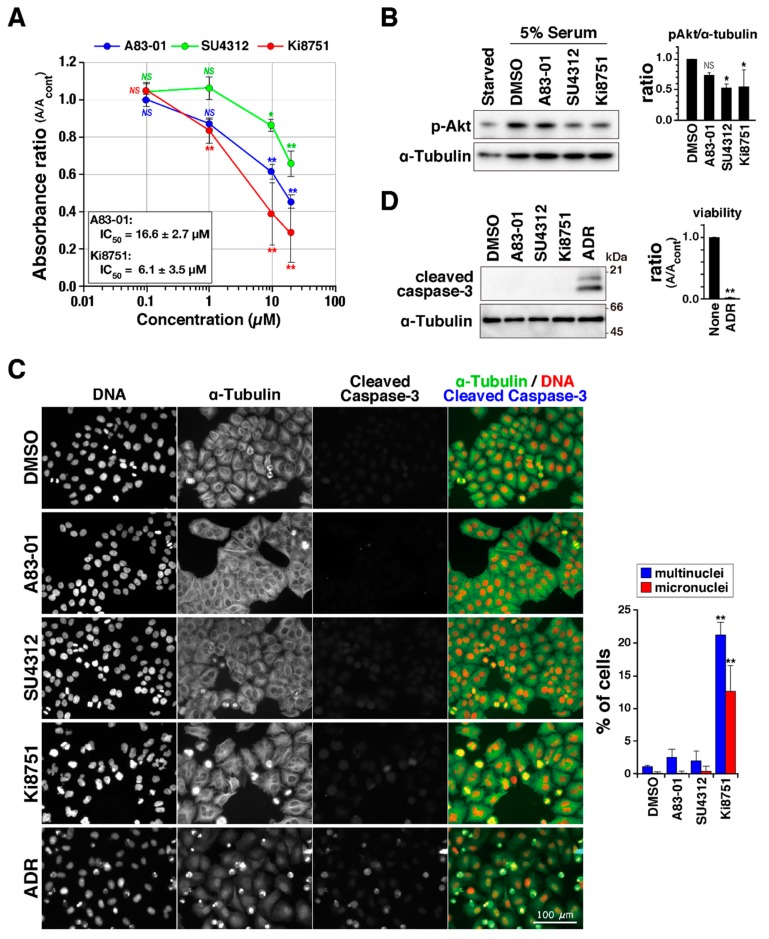
Vascular endothelial growth factor receptor (VEGFR) inhibitors suppress cell proliferation. (**A**) HeLa S3 cells were treated with A83-01, SU4312, and Ki8751 at the indicated concentrations for 2 days, and viable cells were determined by monitoring the absorbance of the formazan at 450 nm. Relative values are shown as a ratio of absorbance to solvent control (di-methyl sulfoxide, DMSO) using the mean ± S.D., calculated from three independent experiments. IC_50_ was calculated in A83-01–, Ki8751–treated cells in each experiment, and the mean ± S.D. was shown in the graph. Asterisks indicate statistical significance (* *p* < 0.05; ** *p* < 0.01; NS, not significant), calculated using Scheffe’s F test. (**B**) Cells were cultured without serum for 1 day and then pretreated with the indicated inhibitors or DMSO as a solvent control for 30 min. Then, serum was added into the culture, and cells were continuously treated with inhibitors for 30 min. Whole cell lysates were analyzed using Western blot analysis with anti-phospho-Akt (pSer473) and anti-α-tubulin antibodies. Phosphorylation of Akt was quantified by measuring the signal intensity of the bands. The ratios of signal intensity of phosphorylated band of Akt to that of α-tubulin are shown as the mean ± S.D., calculated from three independent experiments. Asterisk indicates statistical significance (* *p* < 0.05; NS, not significant), calculated using Scheffe’s F test. (**C**) Cells were treated with 20 µM A83-01, 20 µM SU4312, 20 µM Ki8751, and 4 µM Adriamycin (ADR) for 24 h and then fixed and stained for DNA (red), α-tubulin (green), and cleaved caspase-3 (blue). Scale bar, 100 µm. The number of cells with multinuclei or micronuclei was counted and is shown as the mean ± S.D., calculated from three independent experiments (*n* > 155 in each treatment). Asterisks indicate statistical significance (** *p* < 0.01), calculated using Scheffe’s F test. (**D**) (Left), cells were treated with 20 µM A83-01, 20 µM SU4312, 10 µM Ki8751 and 4 µM ADR for 24 h, and the lysate was prepared and analyzed for cleaved caspase-3. (Right), cells were treated with 4 µM ADR for 48 h, and viable cells were determined as shown in (A). Relative values are shown as a ratio using the mean ± S.D., calculated from three independent experiments. Asterisks indicate statistical significance (** *p* < 0.01), calculated using Student’s *t*-test.

To study the effect of VEGFR inhibitors on cell proliferation, HeLa S3 cells were cultured in the presence of the VEGFR inhibitors Ki8751, SU4312, and A83-01, and cell numbers were estimated using WST-8 (Cell Counting Kit-8). The absorbance of reduced 2-(2-methoxy-4-nitrophenyl)-3-(4-nitrophenyl)-5-(2,4-isulfophen-yl)-2H-tetrazolium monosodium salt at 450 nm showed that the VEGFR inhibitors reduced cell numbers in a concentration-dependent manner (Figure 1A). Inhibition of VEGF/VEGFR signaling was verified by examining the phosphorylation of Akt at Ser473, which is known to occur downstream of VEGFR activation [5]. Although serum-starved cells did not show Akt phosphorylation, addition of serum to the medium after starvation triggered Akt phosphorylation at Ser473 (Figure 1B and Figure S1), validating the specificity of the antibody. Quantification of phosphorylated bands of Akt confirmed that the VEGFR inhibitors partially inhibited VEGFR signaling. To determine whether the reduction of cell viability caused by VEGFR inhibitors could be attributed to cell death via apoptosis, caspase-3 cleavage was examined by immunofluorescence staining and Western blot analyses. In contrast to Adriamycin (ADR)-treated cells, in which almost all cells were dead (Figure 1D, right), the level of the cleaved caspase-3 signal in VEGFR inhibitor-treated cells was not increased (Figure 1C,D and Figure S1), suggesting that the VEGFR inhibitors reduced cell number independent of apoptosis induction at the dosages and treatment times used here. Notably, 10 µM Ki8751-treated cells exhibited nuclear shape changes, including binuclei and micronuclei (Figure 1C). These aberrant nuclei are sometimes observed in cells that prematurely exit mitosis with chromosome segregation errors. Additionally, binucleation is a hallmark for cell abscission defects, raising the possibility that, at the concentrations used here, the VEGFR inhibitors could suppress cell proliferation by causing aberrant cell division.

**Figure 2:**
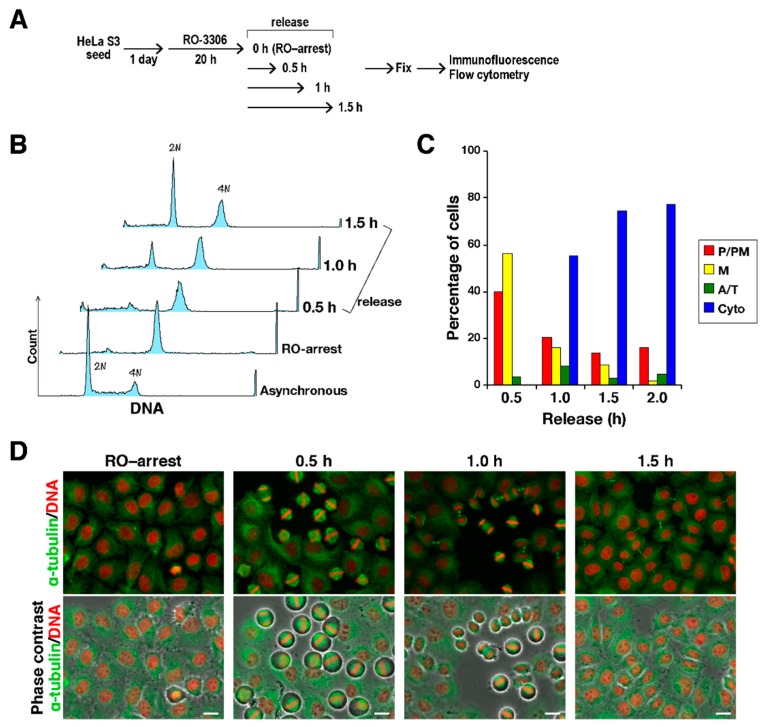
Cell cycle synchronization in M phase. Cells were treated with 6 µM RO-3306 for 20 h. After release from RO-3306 treatment, the cells were fixed at 0.5, 1, and 1.5 h after the release. (**A**) A schematic depiction of the experiment is shown. (**B**) Cells were stained for DNA with propidium iodide, and DNA content was measured by a flow cytometer. Each curve represents 5000 cells. Peak haploid genome equivalents (2N, 4N) are indicated. (**C**) On the basis of α-tubulin and DNA morphologies under a microscope, the M phase cells were classified into four groups: prophase/prometaphase (P/PM), metaphase (M), anaphase/telophase (A/T), and cytokinesis (Cyto). The percentages of cells in each group are plotted (*n* > 204). (**D**) Representative images are shown; α-tubulin (green), DNA (red). Scale bars, 20 µm.

**Figure 3:**
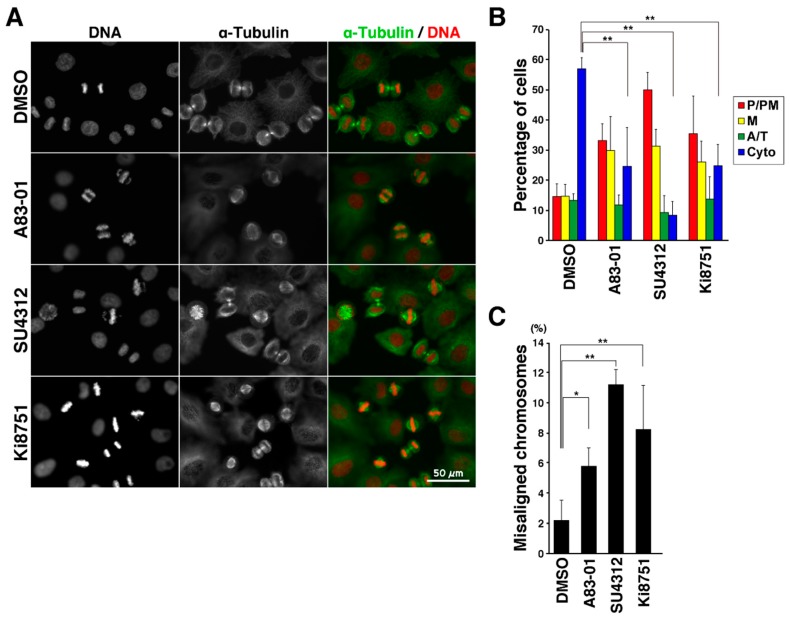
VEGFR inhibitors delay M phase progression. Cells were treated with 6 µM RO-3306 for 20 h. After release from RO-3306 treatment, the cells were incubated with inhibitors for 60 min and fixed with 4% formaldehyde. The fixed cells were then stained for α-tubulin and DNA. (**A**) Representative images are shown. Scale bar, 50 µm. (**B**) On the basis of α-tubulin and DNA morphologies under a microscope, the M phase cells were classified into four groups: prophase/prometaphase (P/PM), metaphase (M), anaphase/telophase (A/T), and cytokinesis (Cyto). The percentages of cells of each group are plotted as the mean ± S.D., calculated from three independent experiments (*n* > 241 in each experiment). (**C**) The number of cells with misaligned chromosomes was counted under a microscope. The percentages of cells exhibiting misaligned chromosomes are plotted as the mean ± S.D. of three independent experiments (*n* > 241 in each experiment). The Tukey–Kramer multiple comparisons test was used to calculate *p* values. * *p* < 0.05; ** *p* < 0.01; NS, not significant.

To explore the effect of the VEGFR inhibitors on M phase progression, HeLa S3 cells were synchronized using the Cdk1 inhibitor RO-3306 [16]. When cells were treated with RO-3306 for 20 h, cells were arrested with 4N DNA content. After release from this arrest by washing cells with PBS(+), cells were incubated on drug-free medium (Figure 2A). Flow cytometry showed that cells with 4N DNA content decreased, and cells with 2N DNA content increased with time after release, indicating that G2-arrested cells entered M phase and progressed to next cell cycle after cell division (Figure 2B). Consistent with this, classification of M phase cells into four categories, namely, prophase/prometaphase (P/PM), metaphase (M), anaphase/telophase (A/T), and cytokinesis (Cyto), on the basis of the α-tubulin and DNA morphologies revealed synchronous M phase progression (Figure 2C,D). When cells were treated with the VEGFR inhibitors after release from RO-3306 treatment, classification of M phase cells showed that most of the control cells had progressed to cytokinesis; however, inhibitor-treated cells were largely inhibited from timely mitotic exit (Figure 3A,B; P/PM, M). In addition, misaligned chromosomes were observed upon VEGFR inhibitor treatment (Figure 3C). These results suggest that treatment of cells with the VEGFR inhibitors delayed M phase progression.

**Figure 4:**
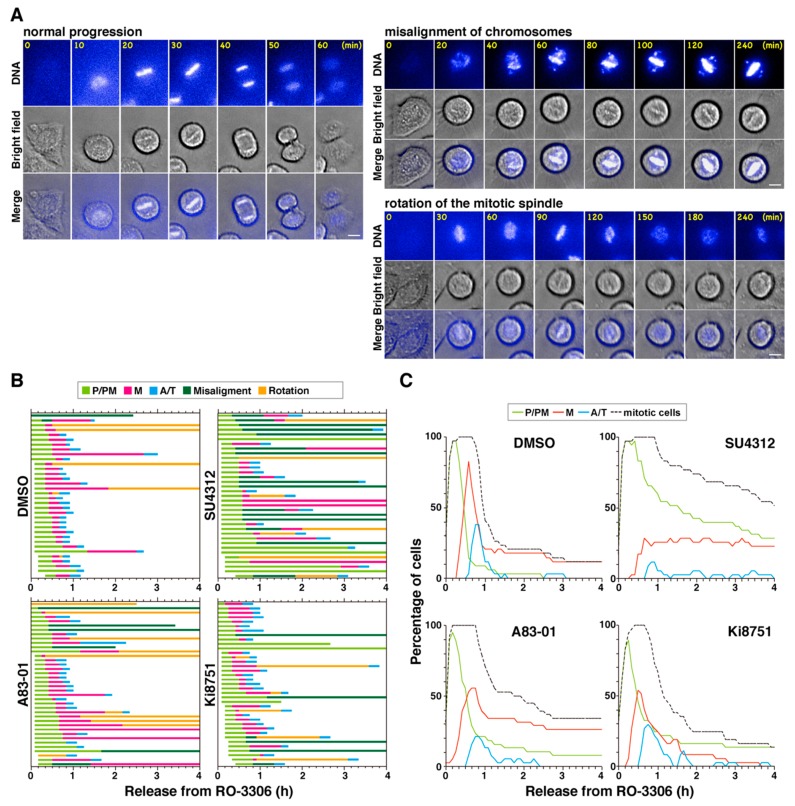
VEGFR inhibitors cause aberrant M phase progression. Cells were treated with 6 µM RO-3306 for 20 h and released in the presence of DMSO, 3 µM A83-01, 10 µM SU4312, or 1 µM Ki8751, together with 0.1 µM Hoechst 33342 to visualize DNA. Mitotic progression was monitored every 5 min for 3 h by time-lapse imaging. (**A**) Representative images of cells exhibiting normal M phase progression, misalignment of chromosomes, and rotation of the mitotic spindle are shown. Scale bars, 10 µm. (**B**) Based on the time-lapse images shown in (**A**), the duration of each mitotic phase (prophase and prometaphase(P/PM, light green), metaphase (M, red), anaphase and telophase (A/T, blue)), misalignment of chromosomes (deep green), and rotation of the mitotic spindle (orange) in individual cells are shown (DMSO, *n* = 34; A83-01, *n* = 38; SU4312, *n* = 35; Ki8751, *n* = 37). (**C**) The percentages of mitotic cells (black), cells in prophase and prometaphase (P/PM, green), metaphase (M, red), and anaphase and telophase (A/T, blue) at the indicated times are shown.

**Figure 5:**
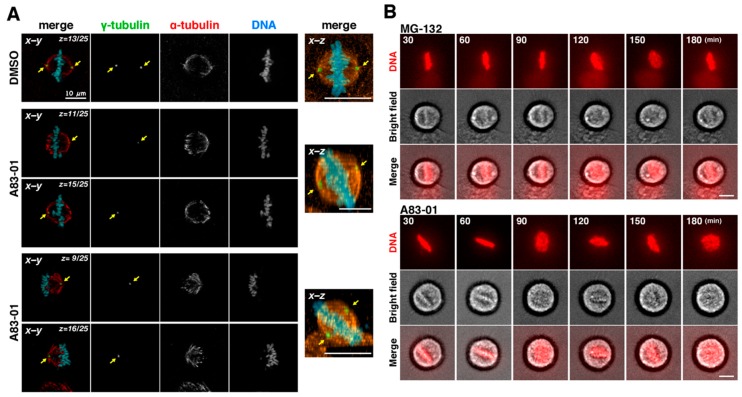
Rotation of mitotic spindle in A83-01-treated cells. (**A**) After incubation with RO-3306 for 20 h, cells were released in the presence of 3 µM A83-01 for 1 h, fixed, and stained for α-tubulin, γ-tubulin and DNA. (Left), *z*–stack images were acquired by using a confocal microscopy, and one or two focal planes (*x*-*y* images) were shown. (Right), the *x*–*z* projections from the *z*–stack images of 25 focal planes (1 µm apart). Arrows indicate the positions of centrosomes. Scale bars, 10 µm. (**B**) After incubation with RO-3306, cells were released in the presence of 0.1 µM Hoechst 33342 with 10 µM MG-132 or 3 µM A83-01. MG-132 or A83-01 was added into the culture at the time of release or at 30 min after the release, respectively. Then, mitotic progression was monitored every 5 min by time-lapse imaging until 3 h after the release. Fluorescence of Hoechst 33342 and bright field images are shown. Scale bars, 10 µm.

To analyze the precise effect of the VEGFR inhibitors on cell division, HeLa S3 cells were synchronized to M phase by incubating the cells with RO-3306, followed by release from the RO-3306 treatment. M phase progression was observed by time-lapse imaging in the presence of Hoechst 33342 to observe DNA (Figure 4A). In control cells, chromosomes were aligned at the cell equator and segregated toward opposite poles. The cleavage furrow ingressed, resulting in the formation of two daughter cells (Figure 4A, normal progression). When cells were treated with the VEGFR inhibitors, misaligned chromosomes were frequently observed (Figure 4A, misalignment of chromosomes); even when most chromosomes were aligned at the cell equator, some chromosomes remained around the poles. In addition, after chromosomes were aligned at the cell equator, they then appeared to disperse again in some cells (Figure 4A, rotation of the mitotic spindle). However, careful observation of these cells under a microscope showed that the chromosomes were not in fact dispersed but that the spindle axis was not parallel to the optical section. It was revealed by γ-tubulin staining that the two poles were located on different focal planes (Figure 5A), suggesting that the aligned chromosomes were misoriented. The VEGFR inhibitors may therefore cause rotation of the mitotic spindle.

M phase progression is divided into three steps: P/PM, M, and A/T, and the time taken for each cell to complete this progression is shown in Figure 4B. Two aberrant phenotypes are also indicated: misalignment of chromosomes (deep green) and rotation of the mitotic spindle (orange). Most control cells completed cell division within 90 min (~80%, *n* = 34). Treatment of cells with the VEGFR inhibitors consistently delayed M phase progression; 53%, 82%, and 30% of cells took more than 90 min to complete cell division after treatment with A83-01, SU4312, and Ki8751, respectively. In approximately 85% of control cells, chromosomes were aligned at the cell equator within 30 min from mitotic entry, as shown by the light green bars in Figure 4B. In contrast, alignment took more than 30 min or failed altogether in 39%, 71%, and 30% of cells treated with A83-01, SU4312, and Ki8751, respectively, suggesting that chromosome alignment was affected by these inhibitors. Although misalignment of chromosomes was observed in only 6% of control cells, it was observed in 16%, 46%, and 14% cells treated with A83-01, SU4312, and Ki8751, respectively. In addition, the number of cells exhibiting spindle rotation was increased by inhibitor treatment from 12% in control cells to 26% and 20% after treatment with A83-01 and SU4312, respectively. These results suggest that the delay in M phase progression was due to a failure in chromosome alignment and spindle rotation.

The line graphs shown in Figure 4C were created based on the results of time-lapse imaging. Compared with the metaphase peak (red line) in control cells, the metaphase peaks were lower in inhibitor-treated cells, confirming that inhibitor treatment partially blocked chromosome alignment. Furthermore, the ratio of metaphase cells did not decrease with time upon A83-01 and SU4312 treatment, which is in agreement with the A83-01- and SU4312-treated cells exhibiting spindle rotation and metaphase arrest. These results suggest that chromosome misalignment and spindle rotation were the causes of M phase delay due to VEGFR inhibitors.

To investigate whether rotation of mitotic spindle was caused by M phase arrest per se, the cells were treated with the proteasome inhibitor MG-132 to arrest cells at metaphase. MG-132 inhibits cyclin B1 degradation, and thereby inhibits anaphase onset. Time-lapse imaging showed that the spindle axis was slightly tilted in approximately 17% of MG-132-treated, metaphase-arrested cells (Figure 5B, *n* = 30). On the contrary, upon A83-01 treatment, spindle rotation was observed in approximately 56% of MG-132-treated cells (Figure 5B, A83-01, *n* = 23). In these cells, spindle axis was severely tilted more than that in MG-132-treated cells. Therefore, the VEGFR inhibitors caused spindle rotation not through metaphase arrest per se.

**Figure 6:**
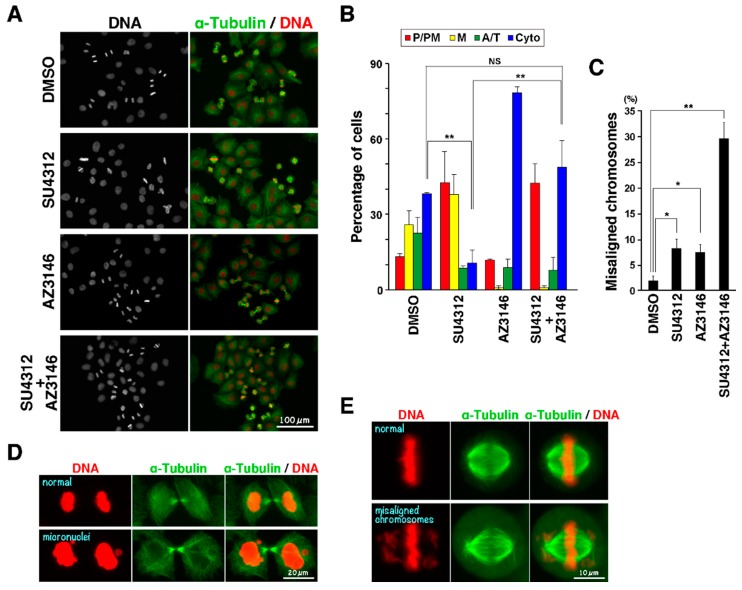
Activation of the spindle assembly checkpoint in SU4312-treated cells. Cells were treated with 6 µM RO-3306 for 20 h. After release from RO-3306 treatment, the cells were incubated with 10 µM SU4312, 2 µM AZ3146, or a combination thereof for 60 min. The cells were fixed with 4% formaldehyde, and stained for α-tubulin and DNA. (**A**) Representative images are shown. Scale bar, 100 µm. (**B**) On the basis of α-tubulin and DNA morphologies under a microscope, M phase cells were classified into four groups: prophase/prometaphase (P/PM), metaphase (M), anaphase/telophase (A/T), and cytokinesis (Cyto). The percentages of cells of each group are plotted as the mean ± S.D., calculated from three independent experiments (*n* > 240 in each experiment). (**C**) The number of cells showing misaligned chromosomes was counted under a microscope, and the percentages are plotted as the mean ± S.D. of three independent experiments (*n* > 240 in each experiment). The Tukey–Kramer multiple comparisons test was used to calculate *p* values. * *p* < 0.05; ** *p* < 0.01; NS, not significant. Representative images of cells with micronuclei and misaligned chromosomes are shown in (**D**) and (**E**), respectively. Scale bars, 20 µm in (**D**), 10 µm in (**E**).

Given that chromosome misalignment and spindle rotation activate the spindle assembly checkpoint (SAC), the M phase delay could be caused by the SAC. To examine this possibility, cells were synchronized with RO-3306 and treated with SU4312 with or without the Mps1 inhibitor AZ3146 [17] during release from RO-3306. Most of the control cells progressed to metaphase or began anaphase (Figure 6A,B, dimethyl sulfoxide (DMSO)). Many SU4312-treated cells failed to completely align their chromosomes at the equator, as shown in Figure 4. In sharp contrast, M phase in AZ3146-treated cells progressed faster than in control cells and chromosomes appeared decondensed, indicating mitotic exit. Similarly, a large number of SU4312-treated cells progressed to cytokinesis upon AZ3146 treatment. These results suggest that the delay in M phase progression in SU4312-treated cells was due to activation of the SAC.

Interestingly, the large number of cells simultaneously treated with SU4312 and AZ3146 still remained blocked in prophase/prometaphase (Figure 6B, P/PM). It has been reported that AZ3146 reduces the kinetochore recruitment of centromere protein E (CENP-E) and thereby inhibits chromosome alignment. Consistent with this, AZ3146 alone caused misalignment of chromosomes (Figure 6C). Given that the VEGFR inhibitors inhibited the chromosome alignment as shown in Figure 6E with a distinct mechanism from that for AZ3146, combination of both agents may cause severe effects on the chromosome alignment. Indeed, this combination generated micronuclei (Figure 6D) and misaligned chromosomes (Figure 6C,E). If AZ3146 at the concentration used here was not able to completely inhibit Mps1 kinase activity, AZ3146 treatment may not override the SAC in cells with severe defects in chromosome alignment, and these cells may remain blocked before anaphase onset. Collectively, a combination of VEGFR inhibitors used here and the Mps1 inhibitor may cause chromosomal instability through induction of misalignment of chromosomes and inactivation of SAC.

## 3. Discussion

In the present study, we found that three VEGFR inhibitors, A83-01, SU4312, and Ki8751, delayed M phase progression, accompanied by misalignment of chromosomes and rotation of the mitotic spindle, at concentrations around the IC_50_. Because cleaved caspase-3 was not detected by immunofluorescence and Western blot analyses when cells were treated with these inhibitors, the resulting suppression of cell proliferation may have been caused not by apoptosis, but by induction of M phase delay. This is a novel mechanism underlying the suppression of cell proliferation by VEGFR inhibitors.

The SAC is able to arrest M phase progression by inhibiting APC/C via mitotic checkpoint complex (MCC) formation when not all kinetochores correctly connect with microtubules emanating from the two opposite poles [18]. Time-lapse imaging analysis in the present study showed that treatment with the inhibitors caused misalignment of chromosomes and rotation of the mitotic spindle. It was shown by γ-tubulin staining of cells treated with VEGFR inhibitors that the two spindle poles were located on different focal planes, confirming rotation of the mitotic spindle. Aberration of the mitotic spindle that causes misalignment of chromosomes and spindle rotation can activate the SAC, which may be the mechanism by which the VEGFR inhibitors delay M phase progression. The SAC-promoting kinase Mps1 phosphorylates Knl1, a component of the KMN network, creating docking sites for SAC proteins and supporting MCC assembly [18]; Mps1 is an essential component of the SAC. Therefore, inhibition of Mps1 prevents SAC activation and induces premature mitotic exit, even when unattached or incorrectly attached kinetochores are present. In the present study, the delay in M phase progression caused by the VEGFR inhibitor SU4312 was counteracted by treatment with the Mps1 inhibitor AZ3146. In this experiment, many cells showed aberrant nuclei, such as micronuclei, which are characteristic of cells that prematurely exit mitosis in the presence of misaligned chromosomes. This confirms that the VEGFR inhibitors may inhibit cell proliferation not by induction of apoptosis, but by SAC-induced M phase delay.

Interestingly, cells with multinuclei and micronuclei were frequently observed in Ki8751-treated cells (Figure 1C), indicating that cells exited mitosis without proper chromosome segregation. Chromosome alignment took more than 30 min or failed in 30% of Ki8751-treated cells (Figure 4B), and approximately 60% of cells had still not proceeded to anaphase at 60 min after the release from RO-3306 treatment (Figure 3B), suggesting that Ki8751 treatment causes delay in chromosome alignment. However, considering that only 30% of Ki8751-treated cells took more than 90 min to complete cell division (Figure 4B,C), the M phase delay may be not sustained. Given that misalignment of chromosomes activates the SAC and delays the M phase progression, Ki8751 may also inhibit the SAC, resulting in generation of cells with multinuclei and micronuclei through failures in cytokinesis of cells with incomplete chromosome segregation. Because this phenotype was observed in only Ki8751-treated cells, a target responsible for this phenotype may be not affected by two other inhibitors.

Inhibitor treatment has an advantage over knockdown experiments; sufficiently reducing protein levels using siRNA usually takes several days, which means that the knockdown effects on gene expression during interphase cannot be excluded. Thus, we used inhibitors to identify target molecules that play roles in M phase progression. Inhibitors were added to cell cultures upon release from cell cycle arrest at the G2/M border, which was induced by the Cdk1 inhibitor RO-3306. While cells were in interphase at the time of release from the arrest, they entered M phase only 10–15 min thereafter. Therefore, the effects of gene expression in interphase can be excluded, indicating that the VEGFR inhibitors studied here affect M phase directly.

In general, small-molecule inhibitors have off-target effects. Ki8751 and SU4312 are selective inhibitors of VEGFR2. However, Ki8751 also inhibits c-Kit and PDGFR tyrosine kinases, albeit with lower potency [14], and SU4312 inhibits PDGFR as well as EGFR, HER-2, and IGF-1R tyrosine kinases with similar potency when used as an E/Z isomer [11]. Although it inhibits VEGFR, A83-01 was originally developed as a TGFβ inhibitor with similar potency [15]. Although reduction of Akt phosphorylation does not contradict the inhibition of VEGFR, the target of these inhibitors responsible for M phase delay has not yet been identified. To determine the exact targets responsible for M phase delay, we performed siRNA experiments; however, we were unable to reach a conclusion. A combination of RNA interference experiments and inhibitor treatments will provide insights into the targets responsible for VEGFR inhibitor-induced M phase delay.

Prolonged activation of the SAC is a mechanism by which cell death may be induced by microtubule-targeting agents, which are extensively used in clinical settings. Cyclin B1 level is a key factor in determining cell fate by regulation of Cdk1 activity; a rapid decrease in cyclin B1 induces mitotic exit without cell death. When caspase-9 is activated via Cdk1 inactivation prior to mitotic exit, cell death occurs [19,20]. This implies that premature mitotic exit prevents mitotic cell death. In the present study, the VEGFR inhibitors caused misalignment of chromosomes and rotation of the mitotic spindle, resulting in M phase delay. Addition of the Mps1 inhibitor AZ3236 caused premature mitotic exit. If cell death is caused by prolonged M phase arrest by treatment with VEGFR inhibitors, premature mitotic exit could counteract the anticancer effects of the VEGFR inhibitors. Very recently, we reported that v-Src induces mitotic slippage by phosphorylating the inhibitory tyrosine residue of Cdk1 [21]. Even when cells were treated with microtubule-targeting agents, they exited mitosis and cell death was inhibited in v-Src-expressing cells. Importantly, knockdown of the C-terminal Src kinase activates Src family kinases and induces mitotic slippage. This implies that cancer cells with increased Src activity may prematurely exit mitosis by overwhelming the SAC. Therefore, cancer cells with activated Src kinases may exhibit resistance to VEGFR inhibitors used in chemotherapy via activated Src-induced premature mitotic exit.

Notably, premature mitotic exit causes asymmetrical chromosome segregation, leading to chromosomal instability (CIN). Low CIN generates genetic diversity and drives cancer genome evolution. This suggests that VEGFR inhibitors may generate genetic diversity and drive cancer genome evolution via induction of aberrant M phase progression. However, higher CIN can lead to cell death and tumor suppression [22,23]. Therefore, in combination with VEGFR inhibitors, agents that induce premature mitotic exit may cause cell death through increasing CIN. These agents include inhibitors of Mps1 and Aurora B kinases, since these kinases are essential for the SAC. If activated Src-induced premature mitotic exit is comparable with that by inhibitors of Mps1 and Aurora B kinases, VEGFR inhibitors may be useful for chemotherapy in cancer cells with activated Src. Further study of combinations of these drugs in various cancer cells could provide a new strategy for cancer chemotherapy.

## 4. Materials and Methods

### 4.1. Cells

HeLa S3 (Japanese Collection of Research Bioresources, Osaka, Japan), human cervix adenocarcinoma, were cultured in Dulbecco’s modified Eagle’s medium (DMEM) containing 20 mM HEPES-NaOH (pH 7.4) and 5% fetal bovine serum (FBS) in 5% CO_2_ at 37 °C.

### 4.2. Chemicals

The VEGFR inhibitors SU4312 (Sigma-Aldrich, St. Louis, MO, USA), A83-01 (StressMarq Bioscience Inc., Victoria, BC, Canada), and Ki8751 (Selleck Chemicals, Houston, TX, USA) were used in the present study. To synchronize cells in M phase, the reversible Cdk1 inhibitor RO-3306 (Selleck Chemicals; Tokyo Chemical Industry, Tokyo, Japan) was used. To arrest cells at metaphase, 10 µM MG-132 (Cayman Chemical, Ann Arbor, MI, USA) was used. These inhibitors were dissolved in dimethyl sulfoxide (DMSO).

### 4.3. Antibodies

The following antibodies were used for immunofluorescence (IF) analysis and Western blotting (WB): rat monoclonal anti-α-tubulin (1:800 for IF, 1:1000 for WB; MCA78G, Bio-Rad Laboratories, Hercules, CA, USA), rabbit monoclonal anti-phospho-Akt (Ser473) (1:2000 for WB; D9E, Cell Signaling Technology, Danvers, MA, USA), mouse monoclonal anti-γ-tubulin (1:400 for IF; GTU-88, Sigma-Aldrich), and rabbit polyclonal anti-cleaved caspase-3 (1:500 for IF; 1:500 for WB; Asp175, #9661, Cell Signaling Technology). In terms of secondary antibodies, Alexa Fluor 488- or 555-labeled, donkey anti-rabbit or goat anti-rat IgG (1:400–1:800; Life Technologies, Waltham, MA, USA) was used for IF. Horseradish peroxidase (HRP)-conjugated donkey anti-mouse IgG (1:4000, 715-035-151), donkey anti-rabbit IgG (1:4000, 711-035-152), and donkey anti-rat IgG (1:4000, 712-035-153) antibodies were purchased from Jackson ImmunoResearch (West Grove, PA, USA) and were used as secondary antibodies for WB.

### 4.4. Immunofluorescence Microscopy

Immunofluorescence staining was performed as described previously [24]. Briefly, cells were fixed with phosphate-buffered saline (PBS) containing 4% formaldehyde for 20 min at room temperature. Then, the cells were permeabilized and blocked with PBS containing 0.1% saponin and 3% bovine serum albumin, followed by incubation with primary and secondary antibodies for one hour each. These antibodies were diluted with PBS containing 0.1% saponin and 3% bovine serum albumin. During the incubation with the secondary antibody, cells were simultaneously incubated with 1 μM Hoechst 33342 for DNA staining. Fluorescence images were captured using a fluorescence microscope (IX-83, Olympus, Tokyo, Japan) equipped with a 20× 0.45 NA and a 40× 0.75 NA objective lens (Olympus). The optical system included a U-FUNA filter cube (360–370 nm excitation, 420–460 nm emission), U-FBNA filter cube (470–495 nm excitation, 510–550 nm emission), and U-FRFP filter cube (535–555 nm excitation, 570–625 nm emission) for observing Hoechst 33342, Alexa Fluor 488, and Alexa Fluor 555 fluorescence, respectively.

Confocal images were captured by using a laser scanning microscope (LSM800; Carl Zeiss, Jena, Germany) equipped with a 63× 1.40 NA oil-immersion objective lens. For observing Hoechst 33342, Alexa Fluor 488, and Alexa Fluor 555, they were excited with the 405, 488, and 561 nm line, and their fluorescence was detected with 400–460 nm, 510–550 nm, and 570–620 nm emission filters, respectively.

Captured images were edited using ImageJ (National Institutes of Health, Bethesda, MD, USA), Photoshop CC, and Illustrator CC software (Adobe, San Jose, CA, USA).

### 4.5. Western Blotting

Cells were solubilized in SDS-sample buffer containing phosphatase inhibitors (50 mM NaF, 20 mM β-glycerophosphate, 10 mM Na_3_VO_4_). Proteins were separated by SDS-polyacrylamide gel electrophoresis and transferred onto polyvinylidene difluoride membranes (PVDF, Pall Corporation, Port Washington, NY, USA). The membranes were blocked with Blocking One (Nacalai Tesque, Kyoto, Japan), and incubated for 1 h at room temperature or overnight at 4 °C with primary and secondary antibodies diluted in tris-buffered saline (TBS) containing 5% Blocking One and 0.1% Tween20. Sequential reprobing of the membranes with various antibodies was performed after inactivation of HRP by 0.1% NaN_3_. Proteins were detected with Chemi-Lumi One L (07880-70, Nacalai Tesque) and Clarity (#1705061, Bio-Rad) using the image analyzer ChemiDoc XRSplus (Bio-Rad).

### 4.6. Cell Cycle Synchronization

For cell cycle synchronization at M phase, the Cdk1 inhibitor RO-3306 was used as described previously [24,25]. Cells were pre-arrested at the G2/M border by treatment with 6 µM RO-3306 for 20 h. After being washed with pre-warmed PBS (37 °C) containing Ca^2+^ and Mg^2+^ (PBS(+)) to prevent detachment of cells at 37 °C on a water bath, cells were incubated in pre-warmed medium containing FBS for 60 min. The cells were fixed in PBS containing 4% formaldehyde at room temperature for 20 min, and then stained for α-tubulin and DNA. Cells were examined under a microscope for mitotic sub-phases and classified into four categories, prophase/prometaphase (P/PM), metaphase (M), anaphase/telophase (A/T), and cytokinesis (Cyto). The percentage of cells in each category among M phase cells was calculated.

Cells in which most chromosomes were aligned at metaphase plate were examined for chromosome alignment, and the number of cells with chromosomes not aligned was counted.

### 4.7. Time-Lapse Imaging

HeLa S3 cells were seeded in a 24-well plate and then cultured with 6 µM RO-3306 for 20 h. After being washed with pre-warmed PBS(+) four times at 37 °C on a water bath, pre-warmed DMEM containing 5% FBS and 0.1 µM Hoechst 33342 was added into the culture with or without the VEGFR inhibitors. Immediately, the 24-well plate was set in the Operetta imaging system (PerkinElmer, Waltham, MA, USA), and live cell images of bright field and fluorescence of Hoechst 33342 were acquired every 5 min for 3 h in a live cell chamber of the Operetta imaging system at 37 °C in 5% CO_2_ [26]. Duration of each category, such as P/PM, M, A/T, ‘misalignment’, and ‘rotation’ was determined. Cells having misaligned chromosome, even when most chromosomes were aligned at metaphase plate, were categorized as ‘misalignment’. Cells with rotating spindle were categorized as ‘rotation’.

### 4.8. Proliferation Assay

Cell proliferation was determined by a Cell Counting Kit-8 (Dojindo, Kumamoto, Japan) according to the manufacturer’s instructions, as described previously [21]. Cells (1 × 10^3^ per well) were seeded in 96-well plates, and the next day, the cells were cultured with SU4312, Ki8751, or A83-01 at 0.1, 1, 10, or 20 µM for 2 days. As a solvent control, cells were cultured in the presence of 0.1% DMSO. On the basis of the absorbance (450 nm) of reduced 2-(2-methoxy-4-nitrophenyl)-3-(4-nitrophenyl)-5-(2,4-isulfophen-yl)-2H-tetrazolium monosodium salt (WST-8), the number of cells was evaluated. To examine the effect of ADR, cells were treated with 4 µM ADR for 2 days, and their number was examined as described above. The absorbance of control cells treated with DMSO or none was set as 1, and the ratio of the absorbance in inhibitor-treated cells to that in control cells was calculated.

### 4.9. Flow Cytometry

To analyze M phase synchronization, cells were treated with RO-3306 for 20 h and then released by washing cells with PBS(+). At 0.5 h after the release, mitotic cells were collected by mitotic shake-off and continuously incubated in polypropylene tubes at 37 °C. At 0.5, 1, and 1.5 h after the release, cells were fixed with 70% ethanol at −30 °C for 1 h. The cells were stained for DNA with propidium iodide in the presence of 200 µg/mL RNaseA at 37 °C for 30 min. DNA contents were analyzed using a flow cytometer (Accuri C6 Plus, BD Biosciences, San Jose, CA, USA). Dead cells were excluded by gating on forward-scatter and side-scatter profiles.

### 4.10. Statistics

Statistical significance was determined with the Statcel add-in program for Microsoft Excel (OMS Publishing, Tokorozawa, Japan) by using results obtained from more than three independent experiments. The Bartlett test was used to determine homogeneity of variance. For analysis among groups with equal variance, data were analyzed by one-way ANOVA and then by the Tukey–Kramer multiple comparisons test. For analysis among groups with unequal variance, data were analyzed by Kruskal-Wallis, followed by the Scheffe’s F test. A *p* value less than 5% was considered to be statistically significant. The statistical outlier was not excluded from the analysis.

## Supplementary Materials

Supplementary materials can be found at http://www.mdpi.com/1422-0067/19/12/4014/s1.

## Abbreviations

CIN	Chromosomal instability
DMEM	Dulbecco’s modified Eagle’s medium
DMSO	Dimethyl sulfoxide
FBS	Fetal bovine serum
IF	Immunofluorescence
HRP	Horse radish peroxidase
MCC	Mitotic checkpoint complex
PBS	Phosphate-buffered saline
RTK	Receptor type tyrosine kinase
SAC	Spindle assembly checkpoint
TBS	Tris buffered saline
VEGF	Vascular endothelial growth factor
VEGFR	Vascular endothelial growth factor receptor
WB	Western blotting
