# Supplementary figures and images for: Inhibitors of the VEGF Receptor Suppress HeLa S3 Cell Proliferation via Misalignment of Chromosomes and Rotation of the Mitotic Spindle, Causing a Delay in M-Phase Progression
Source: Int J Mol Sci. 2018 Dec 12;19(12):4014. doi: 10.3390/ijms19124014 (PMC6320846; doi:10.3390/ijms19124014)

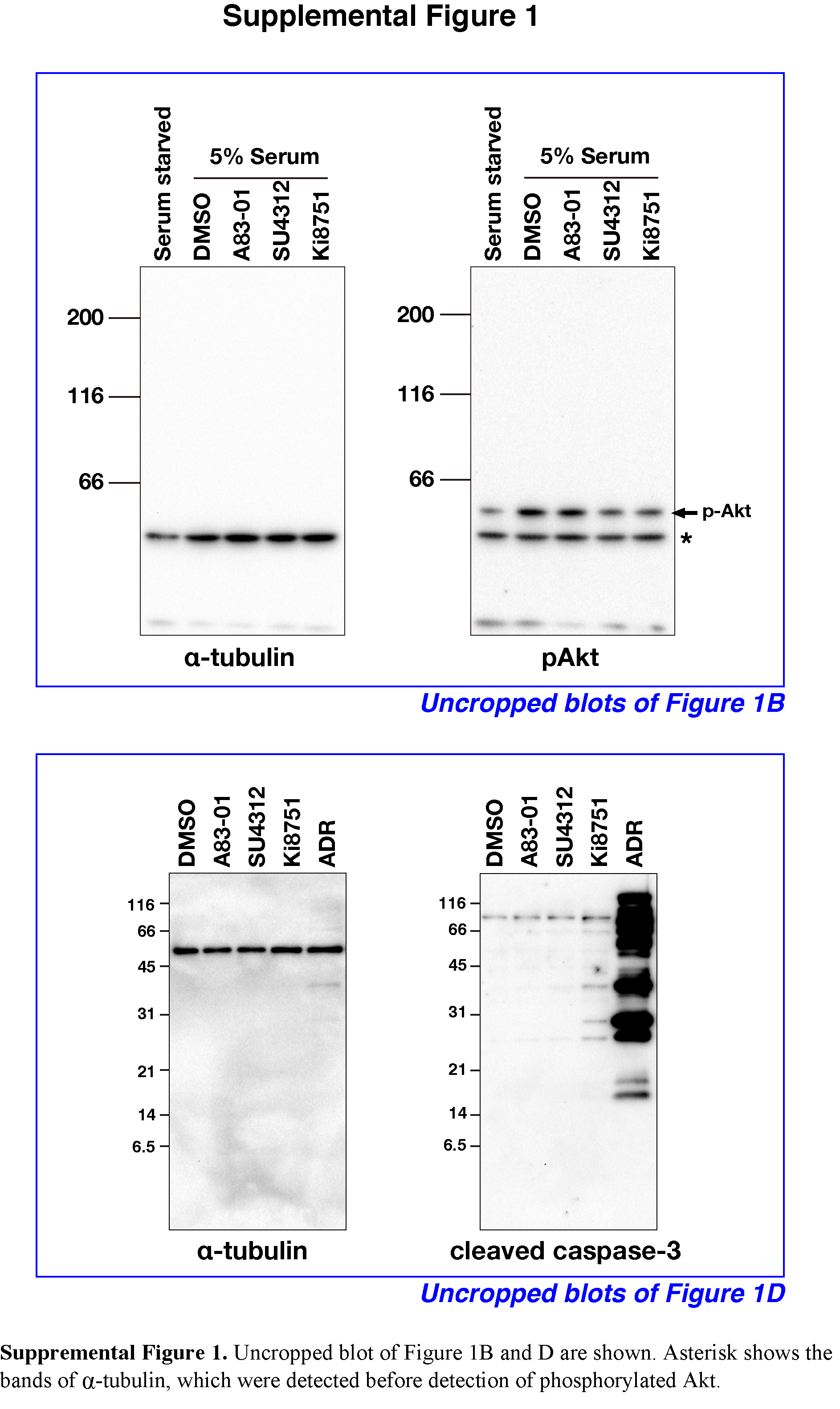

Supplement: Supplementary file 1 [file ijms-19-04014-s001.zip › ijms-381417-supplementary.tif]
